# PSO Algorithm Particle Filters for Improving the Performance of Lane Detection and Tracking Systems in Difficult Roads

**DOI:** 10.3390/s121217168

**Published:** 2012-12-12

**Authors:** Wen-Chang Cheng

**Affiliations:** Department of Computer Science and Information Engineering, Chaoyang University of Technology, 168, Jifong E. Rd., Wufong District, Taichung 41349, Taiwan; E-Mail: wccheng@cyut.edu.tw; Tel.: +886-4-2332-3000 (ext. 5208); Fax: +886-4-2374-2375

**Keywords:** driver assistance system, lane departure warning system, lane following system, lane collision warning system, lane model

## Abstract

In this paper we propose a robust lane detection and tracking method by combining particle filters with the particle swarm optimization method. This method mainly uses the particle filters to detect and track the local optimum of the lane model in the input image and then seeks the global optimal solution of the lane model by a particle swarm optimization method. The particle filter can effectively complete lane detection and tracking in complicated or variable lane environments. However, the result obtained is usually a local optimal system status rather than the global optimal system status. Thus, the particle swarm optimization method is used to further refine the global optimal system status in all system statuses. Since the particle swarm optimization method is a global optimization algorithm based on iterative computing, it can find the global optimal lane model by simulating the food finding way of fish school or insects under the mutual cooperation of all particles. In verification testing, the test environments included highways and ordinary roads as well as straight and curved lanes, uphill and downhill lanes, lane changes, *etc.* Our proposed method can complete the lane detection and tracking more accurately and effectively then existing options.

## 1. Introduction

Lane detection plays an important role in many driver assistance systems such as lane departure warning systems, lane following systems, lane collision warning systems, *etc.*[1–6]. A lane departure warning system mainly determines whether the vehicle is deviating from the current lane or crosses the lane based on the geometrical relationship between the lane and the vehicle. When the vehicle departs from or crosses the lane, a warning is given for the first time to draw the driver’s attention or the turn signal is automatically activated when changing lanes; this can effectively reduce the incidence of accidents. However, the lane following system is usually used to monitor whether the driver is focused on driving or not. When the driver is absent-minded, the vehicle can easily drift to the left and right. Therefore, the driving status can be known through a lane following system. As for the lane collision warning system, if the system does not know the lane location, it might give wrong warnings by mistaking the barriers on the roadside as obstacles in the lane. Therefore, the lane detection can effectively reduce the rate of false alarms. From the above discussion it can be concluded that lane detection indeed plays an important role in driving assistance systems.

The commonly used method in proposed lane detection methods for lane tracking is the Kalman filter [7–15]. It is a high-efficiency recursion filter which can estimate the status of dynamic systems based on a series of incomplete measurements containing interference information. The dynamic system is expressed as a hidden Markov model and its status is used to express the lane models and carry out lane detection and tracking. One of its advantages is its ability to speed up lane detection. It can increase the lane search speed and accuracy in input images since the system establishes the area of interest with lane models. Another advantage is that it can smooth the detection results, reduce the errors and increase the stability of lane detection. However, all these advantages become problems in certain road situations, for example, discontinuous lane markings, lane changes and complicated lane conditions. Since the range of area of interest limits the detection, the above situations can easily cause detection errors. The solution is to use the particle filter (PF) to carry out lane detection and tracking [16–21]. The particle filter is a novel technology for probability-based tracking. It is a method based on the status of dynamic system with multiple Gaussian probability distribution functions, which can still effectively complete lane detection and tracking under conditions such as discontinuous lanes, lane changes and complicated lanes. A particle-based lane detection and tracking approach usually starts with particle prediction, measurement, and re-sampling [1]. Southall and Taylor [16] presented a lane detector based on a condensation framework [22], which uses marking points as measurement features. Each point in the image receives a score based on the distance to the nearest lane marking, and these scores are used to compute the matching score of each particle. The measurement step is considerably simpler than the Kalman filter, because it usually consists of a comparison between the particle and the image data. In addition, many different cues are used in the measurement steps of the particle filter [17–20] so as to increase the accuracy of the measurement steps, for example, edge points, color, template, stereo vision, *etc.* Danescu and Nedevschi [21] used stereo vision to help confirm the lane marking points and used the condensation particle filter to achieve lane detection and tracking. Even so, the particle filter generally takes the particle with the biggest weight or weighted sum of all particles as the last output particle. Therefore, the output result is a local optimal solution rather than a global optimal solution in the problem domain. This paper proposes a new PSO-PF algorithm by combining the particle filter and particle swarm optimization (PSO) method. Such an algorithm is an iterative operation optimization algorithm that further refines the global optimization system status from the current particle filter system status by using the particle swarm optimization method. The particle swarm optimization method takes each particle (status) as an independent individual by simulating the food finding way of fish school or insects, and it can find the global optimal system status under the mutual cooperation of all particles. As a result, PSO-PF can be used to search for the optimal lane model. In such an algorithm, the lane model is firstly defined. Each particle represents a lane model and thus is known as a lane particle. The algorithm can be divided into two stages. In the first stage, the particle filter is used for the prediction, measurement and re-sampling of lane particles. In the second stage, all lane particles in the first stage are taken as the original particles in the particle swarm optimization method and the global optimal lane particles can be obtained through calculation by the particle swarm optimization method.

The remaining sections in this paper are as follows: Section 2 firstly introduces the particle filter and particle swarm optimization method and then specifies the new PSO-PF algorithm (PSO-PF). Section 3 introduces the lane model and how to use PSO-PF to complete the lane detection and tracking. Section 4 shows the experiments and results and the last section presents the conclusions.

## 2. The PSO-PF Algorithm

The PSO-PF algorithm hybridizes a particle filter and a particle swarm optimization algorithm. The section is given over to illustrating the design of this new algorithm.

### 2.1. Particle Filters

Firstly, the dynamic system status within time *t* is expressed as $x_{t}^{i}\in R^{n}$, *i* = 1, 2, …, *N*, wherein, the system status contains the location, speed or range size. The forecasting system of particle filter is as follows:

(1) $$x_{t}=f_{t}\left(x_{t-1},w_{t}\right),$$

where *f_t_* is the system transfer matrix from *R^n^* × *R^n^* → *R^n^*, which contains how to forecast the current status **x***_t_* based on the past status **x***_t_*_–1_. The forecasted noise **w***_t_* is also added into the forecasting process, but the information is independent in time and its distribution is not limited. In the observation status **z***_t_* ∈ *R^p^* at time *t*, **z***_t_* is a column vector of *p*×1, containing the observed status result. The observation system is defined as follows:

(2) $$z_{t}=h_{t}\left(x_{t},v_{t}\right),$$

*h_t_* is the observation matrix from *R^n^* × *R^p^* → *R^n^*, the current status can be evaluated as observation result **z***_t_*, and **v***_t_* is the noise during observation. The main purpose of particle filter is to estimate the probability density function with the observed information {**z**_1_, **z**_2,_…, **z***_t_*}. In such case, *D_t_* is the set of {**z**_1_, **z**_2,_…, **z***_t_*}. Assume the posterior probability distribution function (*pdf*) *p*(**x**_*t*–1_|*D*_*t*–1_) at time *t*−1 is known, the posterior *pdf p*(**x**_*t*_|*D_t_*) can be deduced by Bayesian theorem. This process contains prediction and measurement stages:

(1) *Prediction*: The posterior *pdf p*(**x**_*t*_|*D*_*t*–1_) is propagated at time step *t* using the Chapman-Kolmogorov equation:

(3) $$p\left(x_{t}|D_{t-1}\right)=\int p\left(x_{t}|x_{t-1}\right)p\left(x_{t-1}|D_{t-1}\right)dx_{t-1},$$

wherein *p*(**x***_t_*|**x**_*t*–1_) is a Markov procedure.

(2) *Measurement*: The posterior *pdf p*(**x***_t_*|*D_t_*) is computed using the observation vector {**z**_1_, **z**_2,_…, **z***_t_*}:

(4) $$p\left(x_{t}|D_{t}\right)=\frac{p\left(z_{t}|x_{t}\right)p\left(x_{t}|D_{t-1}\right)}{p\left(z_{t}|D_{t-1}\right)},$$

wherein *p*(**z***_t_*|*D*_*t*–1_) is a normalized item expressed as follows:

(5) $$p\left(z_{t}|D_{t-1}\right)=\int p\left(z_{t}|x_{t}\right)p\left(x_{t}|D_{t-1}\right)dx_{t}.$$

The aim of the PF algorithm is to recursively estimate the posterior *pdf p*(**x***_t_*|*D_t_*). Therefore, this *pdf* is represented by a set of weighted particles $\left\{\left(s_{t}^{i},\pi_{t}^{i}\right),i=1,2,\ldots ,N\right\}$, where the weights $\pi_{t}^{i}\propto p\left(z_{t}|x_{t}=s_{t}^{i}\right)$ are normalized. The final state output of the system **s̄** can be estimated by the following equation:

(6) $$\bar{s}=\sum_{i=1}^{N}\pi_{t}^{i}s_{t}^{i}$$

The basic particle filter algorithm also needs to conduct the particle re-sampling based on the particle weight in addition to the above observation and forecast steps. The particle with higher weight $\pi_{t}^{i}$ has more new particles, and the total number of new particles is equal to that of old particles. Because of different re-sampling methods, many variable algorithms are proposed for the particle filter. The CONDENSATION algorithm firstly proposed by Isard and Blake [22], which is a particle filter algorithm that is easily implemented and thus adopted in this paper.

### 2.2. Particle Swarm Optimization Algorithm

Particle Swarm Optimization (PSO) is a stochastic global optimization technique which has been shown to successfully optimize a wide range of continuous functions [23,24]. The algorithm is based on a flocking of birds or a schooling of fish to look for food. PSO searches a space by adjusting the trajectories of individual vectors, also called particles, which are conceptualized as moving points in multidimensional problem space with two associated vectors, position vector $x_{k}^{i}$ and velocity vector $v_{k}^{i}$, *i* = 1, 2,…, *N* for the current evolutionary iteration *k*. The velocity of the individual particle in PSO is dynamically adjusted according to its own flying experience and its companions’ flying experience flying in the search space. The former was termed cognition-only model and the latter was termed social-only model [25]. Therefore, we get:

(7) $$v_{k+1}^{i}=w\cdot v_{k}^{i}+c_{1}\cdot \mathrm{rand}\cdot \left(x_{\mathrm{pbest}}^{i}-x_{k}^{i}\right)+c_{2}\cdot \mathrm{rand}\cdot \left(x_{\mathrm{gbest}}-x_{k}^{i}\right)),$$

(8) $$x_{k+1}^{i}=x_{k}^{i}+v_{k}^{i},$$

where *w* is inertia weight factor (Hu and Eberhat [26] suggested using 0.4∼0.9), *c*_1_ and *c*_2_ are acceleration constants. *rand* is random number between 0 and 1. $v_{k}^{i}$ is the velocity of particle *i* at iteration *k*. $x_{k}^{i}$ is position of particle *i* at iteration *k*, $x_{\mathrm{pbest}}^{i}$ is the best previous position of particle *i* and **x***_gbest_* is the best previous position among all the particles.

### 2.3. The PSO-PF Algorithm

This section will introduce the newly-proposed PSO-PF algorithm. To facilitate the description of algorithm, we rewrite the particle filter and particle swarm optimization method with a pseudo code program. Algorithm 1 shows a pseudo code program of a standard SIR particle filter. The algorithm contains the parameter setting and main loops. The parameter setting contains set **P** of *N* particle statuses and set **E** of estimated values of all particles that changes with time. The main loop contains such steps as prediction, measurement and resample of particle status, among which the resample consists of the weight calculation of each particle status and the position of new particle in re-sampling based on the weight value. Algorithm 2 shows the pseudo code algorithm of particle swarm optimization method, which also contains the parameter setting and main loops. As for the parameter setting, set **P** represents the set of *N* particles, and set **V** represents the set of movement speeds of all particles in the *k*^th^ iteration. Set **P***_pbest_* is the set of recording the optimal position of each particle, and set P*_gbest_* represents the weighted sum of **P***_pbest_* of all current particles or the set of optimal positions so far.

**Algorithm 1.** A pseudo-code for PF algorithm.

**Procedure** ParticleFilter()
**Var**
**P**[1…*N*]: Particle set;
**E**[1…*N*]: Estimate set along time;
*t*: time;
**begin**
*t* = 0;
Initialize(**P**);
**while** (*t* < *T*) **do begin**
Prediction(**P**);
Estimate(**P**, **E**[*t*]);
Resample(**P**, **E**[*t*]);
*t* = *t* + 1;
**end**
**end**

**Algorithm 2.** A pseudo-code for PSO algorithm.

**Procedure** ParticleSwarmOptimization()
**Var**
**P**[1…*N*]: particle set;
**V**[1…*N*]: velocity set along iteration;
**P***_pbest_*[1…*N*]: the best particle set along iteration;
P*_gbest_*: the best particle of all particle set along iteration;
*k*: iteration;
**begin**
*k* = 0;
Initialize(**P**);
**while** (*k* < *K*) **do begin**
ComputePaticleBest(**P**, **P***_pbest_*[*k*]);
ComputeWeightBest(**P***_pbest_*[*k*], P*_gbest_*[*k*]);
ComputeVelocitys(**V**[*k*]);
Update(**P**);
*k* = *k* + 1;
** end**
**end**

As shown in Equation (6), the particle filter generally takes the weighted sum of all particles or the particle with the biggest weight value as the final output. However, both outputs are approximate optimal solutions or local optimal solutions. Thus in this paper, we proposed a new PSO-PF algorithm by combining the particle filter with the particle swarm optimization method. Since the particle swarm optimization method is an algorithm to get the optimal solution, it is used to further refine the global optimal output from all particle statuses after the particle filter re-sampling. Algorithm 3 shows the pseudo code of the PSO-PF algorithm, which firstly initializes all particle statuses and then uses the particle filter to carry out the prediction, measurement and resample of particle status, and then it further searches for the optimal status from particle statuses after re-sampling by the particle swarm optimization method. Later, it copies all particle statuses after the particle filter re-sampling to the particle swarm optimization method to start the iterative computing program. Since the process of the particle swarm optimization method doesn’t cause any impact on all particle statuses of particle filter, it can be conducted in parallel with the algorithm.

**Algorithm 3.** A pseudo-code for PSO-PF algorithm.

**Procedure** PSOAlgorithmParticleFilter()
**Var**
**P**[1…*N*]: Particle set;
**E**[1…*N*]: Estimate set along time;
*t*: time;
**begin** (*Particle Filter*)
*t* = 0;
Initialize(**P**);
**while** (*t* < *T*) **do begin**
Prediction(**P**);
Estimate(**P**, **E**[*t*]);
Resample(**P**, **E**[*t*]);
*k* = 0; (*Particle Swarm Optimization*)
Initialize(**P**’= **P**);
**while** (*k* < *K*) **do begin**
ComputePaticleBest(**P**’, **P***_pbest_*[*k*]);
ComputeWeightBest(**P***_pbest_*[*k*], P*_gbest_*[*k*]);
ComputeVelocitys(**V**[*k*]);
Update(**P**’);
*k* = *k* + 1;
**end**
*t* = *t* + 1;
**end**
**end**

**Figure 1.**
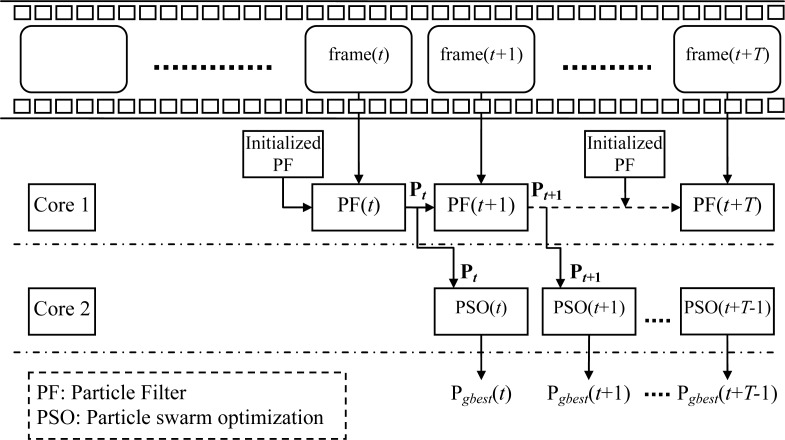
The block diagram for PSO-PF algorithm.

Figure 1 shows the flow chart of *T* video frames continuously executed by the PSO-PF algorithm, in which the upper part shows continuously input video frames and the lower the procedure flow processed by a dual core processor. At time *t*, video frame of frame(*t*) is input and the core 1 firstly initializes the particle state of the particle filter, then predicts, estimates and re-samples particles. Finally, the particle state **P** is output to core 2 as the initialization particle group **P’** of PSO method and the particle state for the next input video frame at time *t*+1. When core 1 is processing the particle filter for video frame of frame(*t+*1), core 2 is executing the PSO method of particle group for video frame of frame(*t*) and outputing the result, P*_gbest_*. Therefore, PSO-PF algorithm can execute parallel processing. In addition, in the execution, if it fails to detect the lane, core 1 will re-initialize the particle state of the particle filter to continue detecting the next input video frame.

## 3. Lane Detection and Tracking Using the PSO-PF

This section illustrates how to use the proposed new PSO-PF algorithm (PSO-PF) to complete the lane detection and tracking. Firstly, it is important to define the status of each particle that represents the lane model which is also identified as the lane particle [21]. With given particles for *N* lanes, then the PSO-PF algorithm is used to complete the prediction, measurement and resample of lane particle as well as search and output the optimal lane particles, respectively, as follows.

### 3.1. Lane Particle

**Figure 2.**
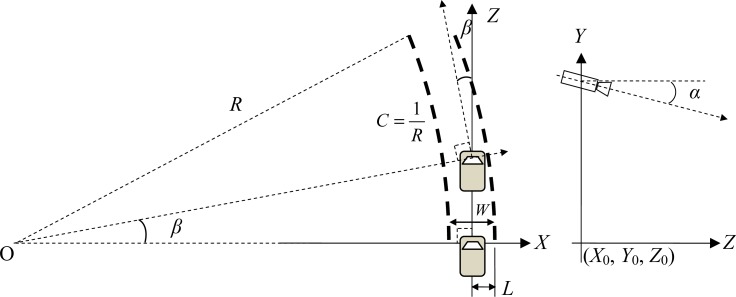
Installation layout of lane models and cameras.

General lane models represent the commonly-used straight line and curve lane models. Although the straight line lane model has a few parameters that are easily calculated, it does not apply to the actual application. Thus we adopted the curve lane model in this paper. The ground lane model defined in this paper is shown in Figure 2.

Given the particles for *N* lanes, the representation vector of each lane particle is shown as follows:

(9) $$x_{i}={\left[C_{i},L_{i},W_{i},\alpha_{i},\beta_{i}\right]}^{T},i=1,\ldots ,N,$$

where *C_i_*, *L_i_* and *W_i_* represent the horizontal curvature, the lateral distance from the vehicle center to the right lane marking and width between the left and right lanes of the *i*th lane particle, respectively. *α_i_* and *β_i_* denote the pitch angle and yaw angle of the camera. Relevant parameters are defined in the left section of Figure 2. The right and left directions of the vehicle are shown in the *X*-axis while the front and back directions are shown in the Z-axis, representing the direction of travel of the vehicle. In the figure, a pair of double, thick dotted arcs represents the position of the lane markings on the ground. The arc curvature *C* is defined as the reciprocal of the radius *R* of the corresponding arc. The right section of Figure 2 shows that the test camera is installed in the vehicle below the rear view mirror of the front windshield which is *H* cm above the ground. The camera faces the front lane of the vehicle and is parallel to the vehicle body. There is a pitch angle *α_i_* with the ground. Based on the assumption of a flat ground, the pinhole camera is used to describe the relationship between the camera coordinate and world coordinate. The pinhole camera model is based on the principle of colinearity, wherein each point on the ground is projected using a straight line from the projection center to the image plane. The origin of the camera coordinate system is at the projection center of the location (*X*_0_, *Y*_0_, *Z*_0_) with respect to the ground coordinate system; the *Z*-axis of the camera frame is perpendicular to the image plane. The rotation is represented using Euler angles *α* (pitch angle), *β* (yaw angle) and *γ* (roll angle) which define a sequence of three elementary rotations around *X*, *Y*, *Z*-axis, respectively. In addition, the direction of the steering wheel and the roll angle are not considered in this paper to simplify the calculation of the lane model.

**Figure 3.**
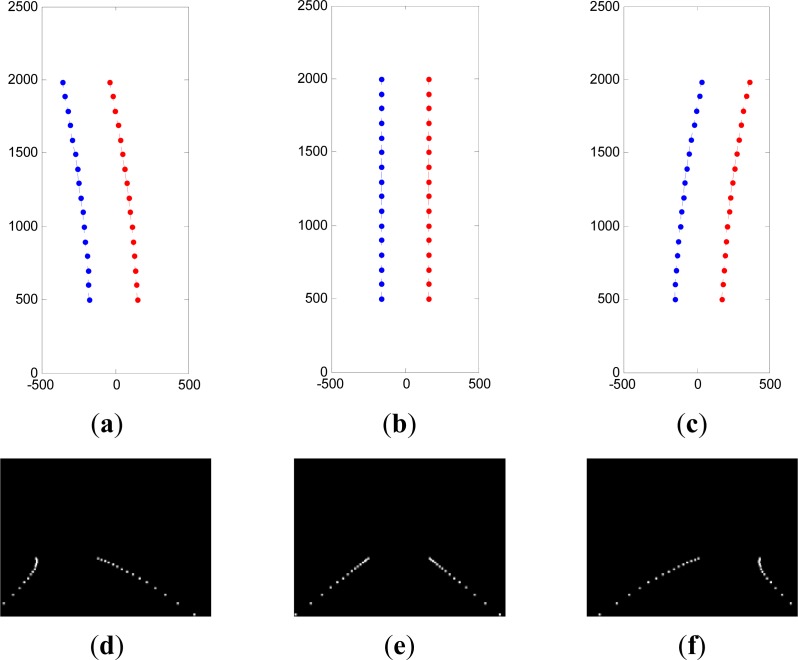
Examples of lane models and corresponding projection images lane model layouts with (**a**) positive curvature, (**b**) zero curvature, and (**c**) negative curvature. (**d**–**f**) are the corresponding projection images of lane models in (a–c).

Figure 3 shows the examples of the lane model and the corresponding projection image. It is assumed that the vehicle is in the middle of the lane, and Figure 3(a–c) represents the ground lane model layout of the left bend (positive curvature = 1e^−5^), straight line (curvature is close to 0) and the right bend (negative curvature = −1e^−5^), respectively. The parallel dotted lines in the figure represent the left and right lane markings (red line for right lane marking, blue line for left lane marking) with a 500 cm to 2,000 cm expression range in front of the vehicle. The interval between the two circles on the dotted line is 100 cm. Figure 3(d–f) shows the images projected by the lane models in Figure 3(a–c) through coordinate transformation, and the bright spots in the images represent the coordinated points at an interval of 100 cm on the ground lane.

### 3.2. Prediction

The purpose of particle prediction is to forecast the current status **x***_t_* based on the past status **x**_*t*–1_. In prediction, the forecasted noise **w***_t_* is introduced; therefore, the particle prediction can be expressed as a simple dynamic motion equation:

(10) $$x_{t}^{i}=Ax_{t-1}^{i}+Bw_{t}^{i},i=1,\ldots ,N,$$

wherein **A** = *diag* (**I**) and **B** = *diag* ([*α*_1_, *α*_2_, *α*_3_, *α*_4_, *α*_5_]). The forecasted noise **w***_t_* can be expressed as a normalized Gaussian noise vector. Since the size of each parameter of the lane particle vector is different, the forecasted noise **w***_t_* vector can be adjusted through the **B** matrix parameters when introduced, so as to make it suitable to the size of each parameter in the lane particle. Using Equation (10), it is easy to predict the next status for each lane particle and to estimate the prediction results.

### 3.3. Measurement

**Figure 4.**
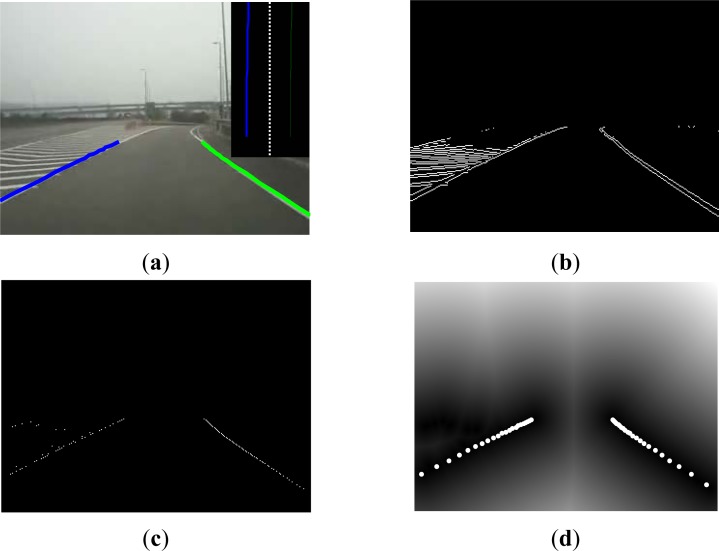
Image measurement examples of lane particle, (**a**) input image, (**b**) edge image, (**c**) lane marking characteristic point image, and (**d**) distance transformation image and lane model coordinate point projection.

The main purpose of the measurement steps is to calculate each lane particle and input the matching degree of lane marking in the images. As mentioned in the above sections, the lane model represented by each lane particle can be expressed as many coordinates on the ground lane markings (as shown in Figure 3(a–c)). These ground coordinate points can be projected on the images through coordinate transformation (as shown in Figure 3(d–f)). Each point in the image receives a score based on the distance to the nearest lane marking and these scores are used to compute the matching score of each lane particle [22]. To complete the measurement steps, we carried out the lane marking detection for input images. Since the lane marking has noticeable edges and characteristics such as high brightness, fixed width and directivity, we not only detected the edge result but also confirmed the position of the lane marking based on the above characteristics. Figure 4 shows an example, in which Figure 4(a) is the input image and Figure 4(b) is the edge image of the bottom half input image. It can be obviously seen that there also exist edges of other painting lines on the ground in addition to the lane edges. As for the lane marking detection method which only considers edges will easily cause wrong detection. Figure 4(c) is the feature image of the input image which is also the detection result of the edge images based on previous characteristics. For easier calculation of the matching degree between the lane particle and lane marking, the distance transformation is finally used to transform the lane marking image into another distance image, as shown in Figure 4(d). It is assumed that the ground coordinate point of the lane model of each lane particle is projected on the image coordinate and can be expressed as (*u_j_*,*v_j_*), *j* = 1,…,*n*, where *n* represents the number of coordinate points as demonstrated by the bright spots in Figure 4(d). The weight value of each lane particle is proportional to the measurement result. Therefore, the weight value of each particle can be expressed as follows:

(11) $$\begin{array}{l} \pi_{t}^{i}=p\left(z_{t}|x_{t}=s_{t}^{i}\right)\\ \;\;\;\;\;\propto p\left(\mathrm{Feature}|x_{t}=s_{t}^{i}\right)=\text{exp}\left[-\frac{\sum_{j=1}^{n}d{\left(u_{j},v_{j}\right)}^{2}}{2n\sigma^{2}}\right],i=1,\ldots N, \end{array}$$

where *d*(*u*, *v*) represents the gray-scale value of coordinates (*u*, *v*) of the distance image, indicating that the distance from the coordinate point to the nearest lane marking. *σ* represents the standard deviation to the Gaussian measurement function.

In the following steps, the weight value of each particle is used to calculate the *cdf* function which is adopted to determine the position of the lane particle in re-sampling. Therefore, more new lane particles can be obtained after re-sampling near the position of the lane particle with bigger weight value; if not, less new lane particles are obtained. As a result, when the lane markings in the input image are continuous, the weight of the lane particle near the lane is big. Most new lane particles after re-sampling gather near the lane, thus the lane detection and tracking can be completed effectively and quickly. However, when the lanes in the input image are discontinuous, the weight of the lane particle near the new lane is small. However, there are still a few corresponding new lane particles after re-sampling. Consequently, after re-adjusting the weight of the lane particle, the lane detection and tracking can still be completed quickly and continuously in the following input images.

### 3.4. Lane Model Refined by PSO

**Figure 5.**
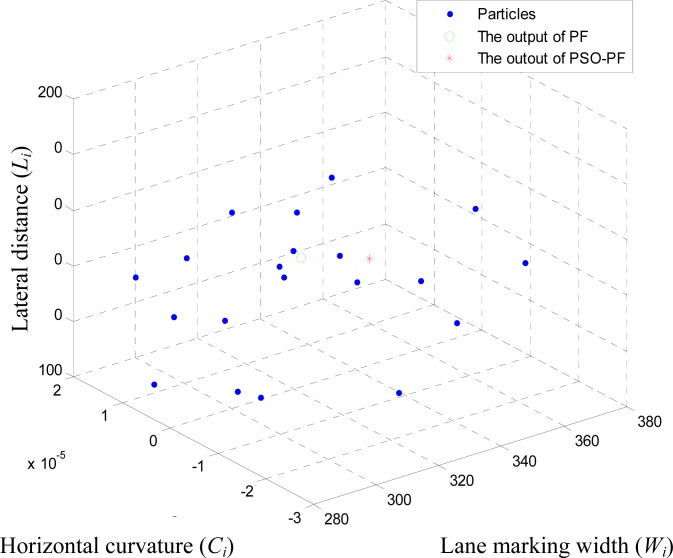
Particle Distribution Example (*N* = 20).

**Figure 6.**
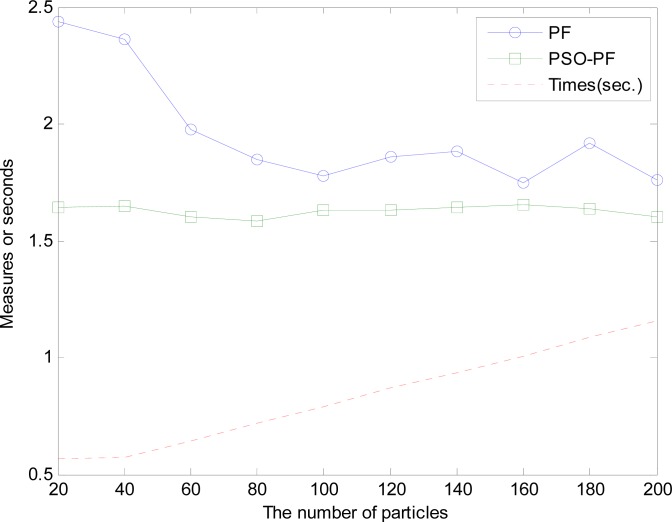
Comparison between particle filter and PSO-PF by using different particle numbers and minimum distances, and relationship diagram of particle filter between different particle numbers and time.

Based on the PSO-PF algorithm, the last step uses the particle swarm optimization method to complete the search for the output global optimal lane particle. Therefore, the algorithm will copy the lane particle of the particle filters as the initial status particle for the particle swarm optimization method. After several iterations, the output global optimal lane particle can be obtained, and it will be used in the follow-up lane deviation or others. Although the iteration of particle swarm optimal method needs extra calculation, the search process with the particle swarm optimization method doesn’t affect the operation of particle filters, thus it can be operated in parallel with the algorithm. Figure 5 shows the particle distribution map corresponding to the input image in Figure 4. To show it clearly, only three parameters of lane particle are shown in the figure, namely horizontal curvature *C_i_*, lateral distance *L_i_* and lane marking width *W_i_*. In this example, the number of particles is 20 and parameters *w*, *c*1, *c*2 are all set to 0.5, 1 and 1, respectively. The blue dots in the figure and green circles represent the particle distribution of particle filters and the corresponding output lane particles respectively. The minimum distance between output lane particle and lane is 2.4759 and the red asterisk means the global optimal lane particle through the PSO-PF algorithm. The minimum distance between the global optimal lane particle and lane is 1.675, thus it is clearly seen that a smaller minimum distance can be obtained through PSO-PF algorithm (see Figure 6 when using 20 lane particles).

## 4. Experimental Results

In this section, we introduce the experimental results and environment. The equipment used in the test included one Intel i5 M480 2.67GHz processor, 4 GB memory and Window 7 operating system while the Matlab software 2009 version was used for development. The test road environment is composed mainly of highways, expressways and ordinary roads, including straight line and curved lanes as well as up and down interchanges. The test process also contains a continuous changing lane. Figure 6 shows the comparison of measured results of output lane particled between the particle filter and PSO-PF using different numbers of particles as well as the diagram of the relationship between the number of different particles of the particle filter and the time consumption. Each test result value is the average value of 100 continuous input images. In Figure 6, the blue circle dotted line and green square dotted line represent the test results of the particle filter and PSO-PF algorithm, respectively. As shown in the figure, if the particle filter is used for lane detection and tracking, the increase of particle number will result in better results, but after the particles are increased continuously, the results will tend to be unchanged. However, when the particle number increases, the required computing time will increase linearly (see the red dotted line in the figure). Therefore, if a higher accuracy is needed, more particles and more time will be required. On the contrary, fewer particles might provide faster processing speed but lower accuracy. The accuracy of PSO-PF algorithm for lane detection and tracking is not affected by the particle number, and different particle numbers are adopted, thus the accuracy of PSO-PF algorithm is higher than that of particle filter. The use of PSO-PF algorithm can get the optimized result with a small number of particles. Since the number of particle used is small, the computing time and memory can also be saved. Although the PSO-PF algorithm needs additional computing time, its particle filter and particle swarm optimization method can be calculated in parallel. When the system is implemented, the actual time won’t be increased. Consequently, the use of PSO-PF algorithm can realize the optimization result by using a few particles, which can effectively improve the efficiency of particle filter for lane detection and tracking. According to experiments, the execution time of particle filter under Matlab environment is about 0.7 sec/frame when using 50 lane particles while that of PSO method is about 0.5 sec/frame. Therefore, under parallel processing for PSO-PF, the bigger value 0.7 sec/frame is taken, by which it can be known that the time required by PSO-PF algorithm is about the execution time of particle filter. In practical operation, PSO-PF algorithm is implemented on a dual-core embedded system with *C* language and running speed of about 15 fps.

**Figure 7.**
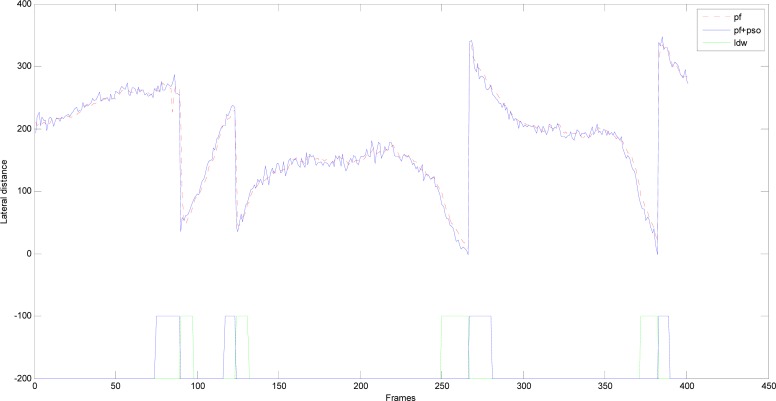
Comparison between particle filter and PSO-PF algorithm on the lateral distance of output lane particles.

**Figure 8.**
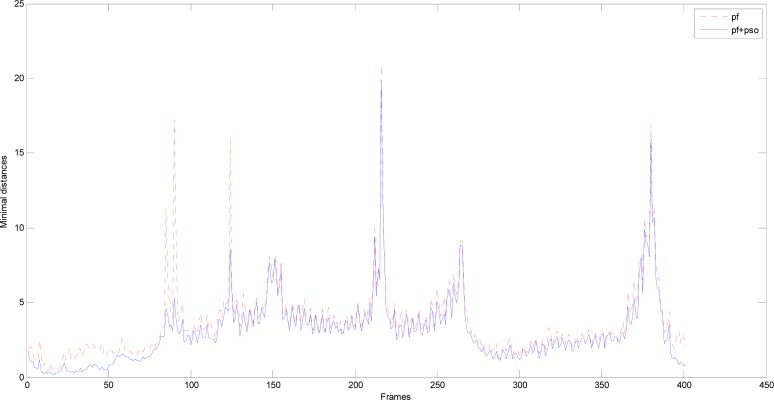
Comparison between Particle Filter and PSO-PF algorithm on the minimum distance of output lane particles.

The output lane particle results of the successively input 800 frames (*i.e.*, the input video images) are shown in Figures 7 and 8, where the number of lane particles is 50. Figure 7 shows the comparison on the lateral distance *L* of the output lane particles between the particle filter and PSO-PF algorithm as the lane detection and tracking (unit: cm). The vehicle line crossing warning signal is also shown in Figure 7. When the left wheel of the vehicle crosses the left lane marking, the left wheel line cross warning signal is given, as shown in the blue square wave below Figure 7. When the right wheel of the vehicle crosses the right lane marking, the right wheel line cross warning signal is given as shown in the green square wave below Figure 7. It can be observed in Figure 7 that four lane changes occurred in the test. The first two are changes from current lanes to left lanes, with the left wheel line cross warning signal occurring firstly followed by the right wheel line cross warning signal. The last two are changes from the current lanes to the right lanes. Therefore, the right wheel line cross warning signal occurs firstly followed by the left wheel line cross warning signal. The duration of the warning signal is related to the exchange speed. In addition, according to Figure 7, the output results of particle filter is smoother than that of PSO-PF algorithm, which is mainly because the output lane particles of particle filter adopt the weighted sum of all lane particles, while those of PSO-PF algorithm adopt the global optimal lane particles. Furthermore, the camera shake also causes obvious changes in the output lane particle results of PSO-PF algorithm due to the high-speed movement of the vehicle.

Figure 8 shows the comparison of the particle filter and PSO-PF algorithm on the minimum distance between output lane particle and lane marking (unit: pixel). It can be clearly found that the minimum distance of the output lane particle by using the PSO-PF algorithm is smaller than that of particle filter, especially when the lane marking is discontinuous or changes. As for frames in the figure with frame numbers less than 125, it can be found that when the vehicle goes into the highway from interchange, the lane marking is shortened and discontinuous, and the frame position changes around 90 and 125 once for each time. The results show that the PSO-PF algorithm can effectively improve the results of the particle filter, although in some circumstances, there is still little difference between our proposed method and particle filter, as frame positions around 265 and 380 show in the figure.

**Figure 9.**
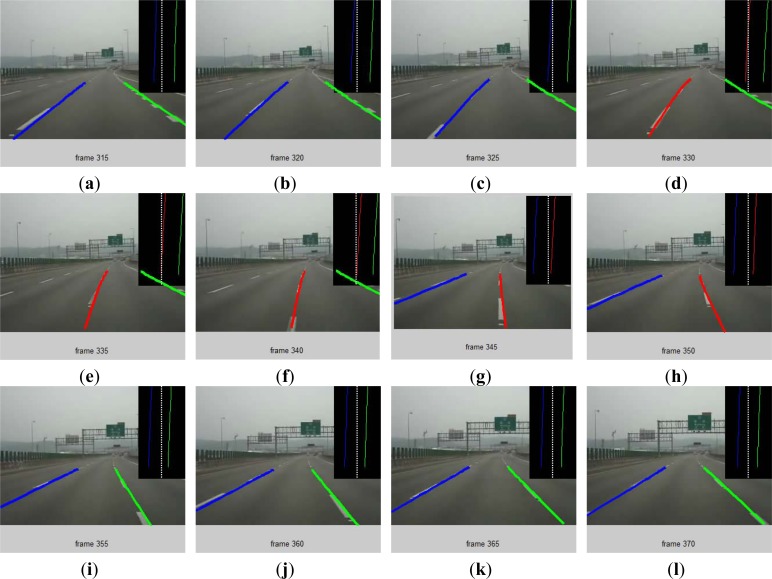
Continuous Images of a lane change example.

Figure 9 shows the continuous image of a lane change process in which vehicle changes from the current lane to the left lane (which corresponds to the second lane change shown in Figure 7). In the figure, an image is displayed every 5 frames. The blue line in the image represents the left lane marking and the green line represents the right lane marking, as shown in Figure 9(a–c). When the left wheel of the vehicle crosses the lane marking, the blue color on the left edge changes to red and the left wheel line cross warning signal is given, as shown in Figure 9(d–f). When the vehicle center crosses the left lane marking, the original left lane edge becomes the right lane marking of the new lane, and the right wheel of the vehicle crosses the right lane marking. Therefore, the right lane marking in the image changes from green to red and the right wheel line cross warning signal is given, as shown in Figure 9(g,h). When the vehicle completes the lane change, the right lane marking in the image turns back to green and the right wheel line cross warning signal is cancelled, as shown in Figure 9(i–l). The original movie files showing the test results can be downloaded from http://www.cyut.edu.tw/~wccheng/.

## 5. Conclusions

In this paper, we have proposed a new algorithm to improve the efficiency of the particle filter, which is known as the particle swarm optimization particle filter (PSO-PF). The algorithm combines the particle filter and the particle swarm optimization method and has the following properties: (1) the algorithm uses the particle swarm optimization method to further search for the global optimal output system status from among the system statuses of the particle filter, therefore, the optimal solution to a problem can be obtained; (2) since each particle filter executed includes the prediction, measurement and resample of the system status, the computation is rapid. While the particle swarm optimization method is an algorithm based on iteration, thus the computing process requires more time, the PSO-PF algorithm uses the particle filter which can compute quickly to search for the local optimal solution or approximate optimal solution, and then uses the particle swarm optimization method to search for the global optimal solution, henceforth, this results in a better efficiency; (3) both the particle filter and particle swarm optimization method are solutions based on the particles, thus they are easy to integrate and implement; (4) since the particle filter and particle swarm optimization method don’t affect each other, they can be computed in parallel to increase the overall calculation speed; (5) as shown in the experimental results, the particle number of the particle filter is proportional to the calculation speed and also to the output accuracy, thus the particle filter can get better output but require more time if more particles are used. On the contrary, if fewer particles are used, less time will be needed for calculation but worse results will be obtained. However, the PSO-PF algorithm can achieve better efficiency while using fewer particles. Finally, we used the PSO-PF algorithm to carry out the lane detection and tracking immediately and then compared its results with those of the particle filter. The experimental results verify that the method can effectively improve the results of the particle filter for lane detection and tracking.
